# Research on SSR Genetic Molecular Markers and Morphological Differences of Different *Pelodiscus sinensis* Populations

**DOI:** 10.3390/genes16030318

**Published:** 2025-03-07

**Authors:** Yixin Liang, Changqing Huang, Pei Wang, Hewei Xiao, Zi’ao Wang, Jiawei Zeng, Xiaoqing Wang, Shuting Xiong, Yazhou Hu, Qin Qin

**Affiliations:** 1 College of Fisheries, Hunan Agricultural University, Changsha 410128, China; liangyixin1218@163.com (Y.L.); hcq22223@163.com (C.H.); gavin_zjw@foxmail.com (J.Z.); wangxiao8258@126.com (X.W.); stxiong@hunau.edu (S.X.); huyazhou@hunau.edu.cn (Y.H.); 2 College of Biology and Environmental Sciences, Jishou University, Jishou 416000, China; wangpei0229@126.com; 3 Hunan Aquatic Foundation Seed Farm, Changsha 410128, China; x42643934@163.com; 4 College of Animal Science and Technology, Hunan Biological and Electromechanical Polytechnic, Changsha 410128, China; 13739069939@163.com

**Keywords:** *P. sinensis*, morphological, SSR, genetic difference

## Abstract

Background/Objectives: The Chinese soft-shelled turtle (*Pelodiscus sinensis*) is an important species in freshwater aquaculture. Genetic admixture and degradation due to rapid industry expansion threaten sustainable development. This study aims to assess the genetic diversity and structure of six *P. sinensis* populations for better management. Methods: We combined morphological analysis and microsatellite markers to evaluate the genetic diversity of six populations. A discriminant function based on morphology was developed, achieving 71.4% classification accuracy. Two SSR markers were identified to specifically distinguish the HS population. Results: The six populations were classified into three subgroups. Frequent gene flow was observed among the CY, W, and DT populations, with most genetic variation occurring within individuals. However, significant genetic differentiation was detected between populations. While gene flow enhanced diversity, it suppressed differentiation. Conclusions: This study provides insights into the genetic structure and diversity of six *P. sinensis* populations. The discriminant function and SSR markers offer a basis for germplasm conservation and management, supporting sustainable aquaculture development.

## 1. Introduction

The Chinese soft-shelled turtle (*P. sinensis*) is an economically important freshwater aquaculture reptile in China [1], widely distributed across East and Southeast Asia [2]. According to the Fisheries Statistical Yearbook of China, the aquaculture production of *P. sinensis* reached 370,000 tons in 2022. Currently, the main species of soft-shelled turtles farmed in China include the native *P. sinensis*, introduced species, newly bred varieties, and hybrid varieties [3]. The native *P. sinensis* is a geographically differentiated subspecies that has gradually evolved through long-term geographical isolation across various regions of China. The introduced species refers to those brought from abroad and selectively bred over multiple generations [4]. New varieties [5] are developed by selecting individuals with beneficial mutations from the native *P. sinensis* population and breeding them through several generations. Hybrid varieties are produced by crossing two populations with a significant genetic distance [6]. With the rapid development of the *P. sinensis* farming industry, the market has become increasingly mixed with native, introduced, newly bred, and hybrid varieties, resulting in genetic degradation and a decline in genetic traits, which severely threatens the sustainable development of the industry. Meanwhile, many farmed *P. sinensis* organisms have escaped into natural waters, and combined with overfishing and the impact of environmental disasters, the population of wild *P. sinensis* has dramatically decreased, leading to a severe loss of genetic resources. In 2000, the wild *P. sinensis* was listed as an endangered species in the International Union for Conservation of Nature (IUCN) Red List of Threatened Species [6]. Understanding the genetic diversity and structure of populations aids in regulating hybrid breeding, preventing overfishing, and conserving germplasm resources, thereby ensuring the sustainable exploitation of the population [7]. However, there is currently limited research on the genetic differences among different populations of the *P. sinensis*, and due to technical limitations, the findings from various studies are often contradictory [8]. Therefore, to standardize the management of *P. sinensis* farming populations and make rational use of their genetic resources, there is an urgent need to analyze the genetic diversity of the main farming populations of *P. sinensis*.

Morphological methods are the most traditional and direct approach for analyzing population genetic variation and have been widely applied in studies on the variation and identification of *P. sinensis* populations. However, since morphological traits are influenced by both genetic factors and external environmental conditions and can be affected by growth stages and data processing methods, they must be combined with genetic molecular markers to objectively evaluate the genetic diversity of populations [9]. With the continuous advancement of high-throughput sequencing technology, molecular marker-based approaches for population genetic analysis and species identification have become increasingly mainstream [10]. Among the various types of molecular markers, single nucleotide polymorphisms (SNPs) and simple sequence repeats (SSRs, also known as microsatellites) are considered the most promising. SNPs refer to polymorphisms in DNA sequences caused by single-base transitions or transversions, resulting in genetic variation. They are characterized by high genetic stability, widespread distribution across the genome, and a typical biallelic nature, making them highly suitable for genetic studies [11]. On the other hand, SSRs are simple tandem repeat sequences uniformly distributed throughout eukaryotic genomes, consisting of 2–6 nucleotide repeat motifs. SSR markers are highly polymorphic, abundant, and reproducible, and they require only small amounts of DNA for analysis [12]. Given these characteristics, researchers can select the appropriate molecular marker based on laboratory conditions and the specific requirements of their study. To date, SSR markers have been widely applied in the analysis of genetic variation and species identification in aquatic animals [13,14,15]. In the evaluation of genetic diversity in *P. sinensis* populations, molecular markers such as mitochondrial genes [6], SSRs [16,17], and SNPs [18] have been used. However, there has been no precedent for studies combining morphological analysis with these methods, and the number of populations studied has been limited, so conducting genetic differentiation analysis between populations based on both phenotypic and genotypic data provides a more comprehensive and reliable assessment of the genetic structure.

Therefore, to objectively evaluate the genetic diversity and genetic structure of *P. sinensis* farming populations and to standardize their management, this study employs both morphological analysis and microsatellite markers to assess the genetic diversity across six different populations and develop germplasm identification markers, providing a reference for scientific breeding of *P. sinensis*.

## 2. Materials and Methods

### 2.1. Sample Information

**Figure 1:**
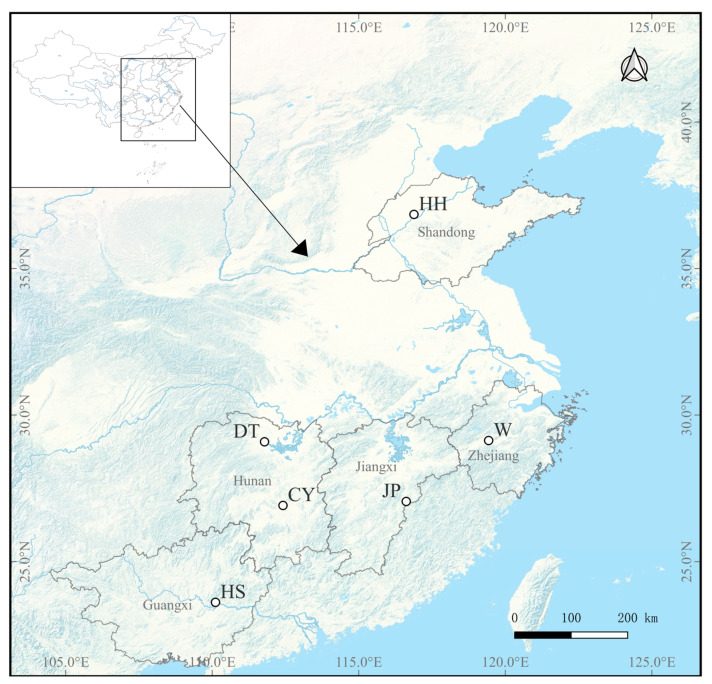
Map shows collection locations of the samples of *P. sinensis* populations from 6 different regions. HH, Yellow River; W, Wu; DT, Dongting; CY, Changyong; JP, Japanese; HS, Huangsha.

Information about the samples and the geographical coordinates of the six different *P. sinensis* populations used in the experiment are presented in Table 1 and Figure 1. The age of each group of *P. sinensis* is one year. All sampled *P. sinensis* populations were obtained from local breeders and were artificially cultured. Age information for each population was provided by the aquaculture farmers who supplied the samples.

### 2.2. Morphological Analysis

**Figure 2:**
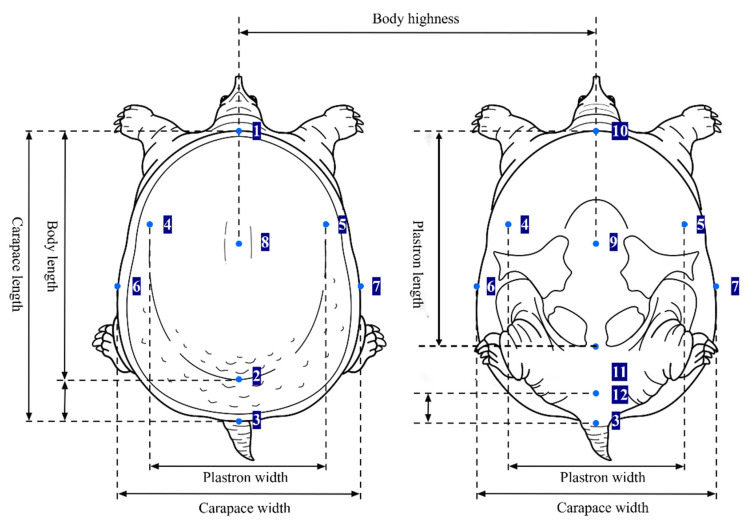
Measurements of the main morphological traits of *P. sinensis*: 1–2, Body Length (BL); 1–3, Carapace Length (CL); 4–5, Plastron Width (PW); 6–7, Carapace Width (CW); 8–9, Shell Height (SH); 10–11, Plastron Length (PL); and 12–3, Back Apron Width (BAW).

According to the Chinese Softshell Turtle National Standard (GB21044-2007) [19], seven morphological traits of six *P. sinensis* populations were measured: Body Length (BL), Shell Height (SH), Carapace Length (CL), Carapace Width (CW), Plastron Length (PL), Plastron Width (PW), and Back Apron Width (BAW). The representation of these morphological traits is shown in Figure 2. These traits were measured with a digital caliper (estimated to 0.01 mm), except for body weight, which was measured with a digital balance (estimated to 0.01 kg). Measurement data were organized using Excel, each morphological parameter is expressed as the mean ± standard deviation, and normality tests of the morphological traits were conducted using SPSS 24.0. To minimize the effect of body weight differences on morphological traits [20], each measured parameter was normalized by dividing by CL (Table 2 and Figure 2), resulting in the following ratios: X_1_ = BL/CL, X_2_ = SH/CL, X_3_ = CW/CL, X_4_ = PL/CL, X_5_ = PW/CL, and X_6_ = BAW/CL. Subsequently, clustering and discriminant analyses were performed using SPSS 24.0, and NMDS analyses were conducted using the OmicShare data analysis platform https://www.omicshare.com/tools (accessed on 7 August 2024).

### 2.3. SSR Marker Development and Screening

The previous sequencing data of our laboratory were used to develop SSR markers (reference number PRJNA1053618), which were identified using MISA v2.0 software by searching for single-nucleotide repeats with a minimum of 10 repetitions; dinucleotide repeats with a minimum of 6 repetitions; trinucleotide repeats with a minimum of 5 repetitions; and tetranucleotide, pentanucleotide, and hexanucleotide repeats with a minimum of 5 repetitions. To enhance the accuracy of the SSRs, the identified SSRs were intersected with InDel markers, selecting those with a variation in at least 5 bases and with InDels located within 5 bp upstream or downstream of the SSR region [21]. A total of 113 SSR loci were then randomly selected for primer design using Primer v3.2.5.0 software. DNA samples were randomly selected and pooled from 10 individuals in each population to create a mixed DNA template for the initial screening of SSR markers. The PCR reaction mixture consisted of 12.5 μL Mix, 1 μL Primer, 1 μL DNA, and 9.5 μL H_2_O, with the following amplification program: 95 °C for 5 min, followed by 35 cycles of 94 °C for 30 s, 56–61 °C for 30 s, and 72 °C for 1 min, with a final extension at 72 °C for 5 min. The PCR products were detected by agarose gel electrophoresis, and primers producing clear single bands were selected. The selected primers were then used for PCR amplification at the optimal annealing temperature, and the amplification products were detected using 8% agarose gel electrophoresis for further screening. Primers showing good polymorphism and clear bands were selected.

The final selection consisted of 12 primers, which were used for fluorescence PCR amplification with DNA samples from all individuals across the six populations as templates. Fluorescent dyes (FAM, ROX, HEX, and TAMRA) were randomly attached to the 5′ end of the primers. The primer information for the SSR markers is provided in Supplementary Table S1. A total of 180 individuals, with 30 *P. sinensis* selected from each population, were sampled for the study. According to the Biomedical Research Ethics Committee of Hunan Agricultural University (Changsha, China), prior to the experiment, the turtles from each population were anesthetized in the laboratory by immersion in MS-222 (Coolaber, Beijing, China) at a concentration of 8000 mg/L for 300 s. DNA was extracted using the Easy-Pure Genomic DNA Kit (Beijing TransGen Biotech Co., Ltd., Beijing, China). The quality and purity of the DNA (OD260 nm/OD280 nm = 1.8–2.0) were assessed using 1% agarose gel electrophoresis and NanoDrop 1000 spectrophotometry (Thermo Fisher Scientific, Waltham, MA, USA). Fluorescence PCR amplification was then performed using the extracted DNA as templates. The amplification products were analyzed via capillary electrophoresis [22].

### 2.4. Genetic Differentiation Analysis

The number of alleles (Na), effective number of alleles (Ne), observed heterozygosity (Ho), expected heterozygosity (He), Shannon’s information index (I), polymorphic information content (PIC), Hardy–Weinberg equilibrium (P_HWE_), inbreeding coefficient (Fis), and genetic differentiation index (F_ST_) were calculated using GenAlEx 6.5 software [23]. Additionally, the Nei’s genetic distance matrix was constructed, followed by principal coordinate analysis (PCoA) and analysis of molecular variance (AMOVA). Gene flow (Nm) between populations was estimated using the following formula: Nm = (1−F_ST_)/4Fst [24]. The PIC values were calculated using Cervus 3.0 software. The Frequency of null alleles (Fna) values were calculated using the Genepop 4.7.5 software. The Nei’s genetic distance matrix was formatted according to the manual and imported into MEGA 11 software [25] to construct the UPGMA phylogenetic tree. The data were then exported from GenAlEx 6.5 in STRUCTURE software format and subsequently imported into STRUCTURE 2.3.4 software [26], with parameters set as follows: Length of Burnin Period = 10,000, number of populations (K) ranging from 2 to 6, with 10 replications per K. The results were compressed and uploaded to an online analysis tool developed by the Institute of Computing Technology, Chinese Academy of Sciences, to compute Delta K values: Ancestry Model select Admixture Model. The website address for this tool is https://lmme.ac.cn/StructureSelector/ (accessed on 18 February 2025). SSR markers suitable for species identification were selected based on the electropherogram results obtained from capillary electrophoresis of the six populations.

## 3. Results

### 3.1. Statistical Analysis and Cluster Analysis of Morphological Traits

**Figure 3:**
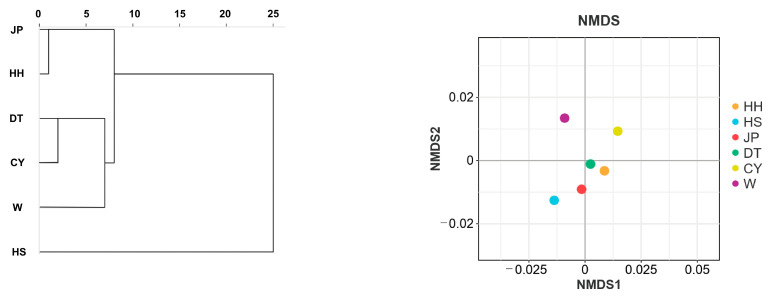
Cluster analysis and NMDS analysis of *P. sinensis* populations based on morphological data. The JP population clustered with the HH population. The DT population clustered with the CY population and then grouped with the W population. Finally, the HS population clustered with the other five populations. The results of the NMDS analysis corroborated the findings of the clustering analysis.

After testing for normality, the morphological data of each population were found to follow a normal distribution. The body weight and morphological trait data for each population are provided in Supplementary Table S2, the proportional parameters of the morphological traits for the *P. sinensis* populations are presented in Supplementary Table S3. A cluster analysis based on the morphological data was conducted on the six *P. sinensis* populations. The results showed that the six populations were divided into three clusters: the HH and JP populations formed one cluster, the HS population formed a separate cluster, and the DT population first clustered with the CY population before forming a cluster with the W population. This clustering result was further validated by NMDS analysis, which showed that the W and CY populations were closest to the DT population; the HH population was adjacent to the JP population; and the HS population was closest to the JP population, first clustering with the JP and HH populations. This outcome strongly supports the conclusions drawn from the cluster analysis (Figure 3).

### 3.2. Stepwise Discriminant Analysis

Based on stepwise discriminant analysis, the six parameters with the greatest contribution were identified, and parameter X_6_ was excluded. A Bayesian discriminant function was established using the selected parameters. After cross-validation, the discriminant accuracy for each population ranged between 56.7% and 90.0%, with an overall accuracy of 71.4%. The discriminant rates for the DT and HH populations were relatively high, at 90.0% and 73.1%, respectively, while the CY population had the lowest discriminant rate at 56.7% (Table 3).

HH = 441.205X_1_ + 311.293X_5_ − 92.829X_2_ + 549.880X_4_ + 266.536X_3_ − 569.608

HS = 408.713X_1_ + 316.080X_5_ − 21.378X_2_ + 449.931X_4_ + 331.142X_3_ − 569.115

JP = 405.014X_1_ + 322.081X_5_ − 74.133X_2_ + 490.591X_4_ + 311.940X_3_ − 567.959

DT = 446.202X_1_ + 303.967X_5_ − 37.950X_2_ + 463.005X_4_ + 285.297X_3_ − 557.338

CY = 463.579X_1_ + 341.898X_5_ − 88.888X_2_ − 466.774X_4_ + 279.832X_3_ − 579.546

W = 469.115X_1_ + 313.838X_5_ − 56.222X_2_ + 511.882X_4_ + 280.815X_3_ − 610.530

### 3.3. Genetic Diversity Analysis

The number of Na at the 12 SSR loci ranged from 1.833 to 12, with an average of 6.972 alleles. The Ne ranged from 1.036 to 5.409, with an average of 3.437. Ho ranged from 0.022 to 0.994, while He ranged from 0.032 to 0.801. The I values ranged from 0.081 to 1.847, with an average of 1.249. The Nm values across the loci ranged from 0.780 to 9.295, with an average of 1.213. Among the 12 SSR loci analyzed, eight loci exhibited a polymorphic information content (PIC) value greater than 0.5, indicating high polymorphism, while three loci showed moderate polymorphism (0.25 ≤ PIC < 0.50), and one locus had a PIC value of less than 0.25. The majority of loci displayed high polymorphism, making them well-suited for the genetic analysis of *P. sinensis* populations.

Regarding gene flow, nine loci exhibited moderate gene flow (1 < Nm < 4), two loci showed weak gene flow (Nm < 1), and one locus exhibited strong gene flow (Nm > 4). Fis ranged from −0.275 to 0.875, with an average of −0.005, suggesting an excess of heterozygotes. Fna ranged from 0.000 to 0.492, indicating the presence of null alleles. Additionally, the Fis values ranged from −0.005 to 0.875, with a mean of −0.005 (Table 4). The Hardy–Weinberg equilibrium analysis (Table 5) revealed that multiple loci in all populations significantly deviated from P_HWE_ (*p* < 0.05). Notably, the W population exhibited significant deviations at nine loci (*p* < 0.05).

The genetic diversity parameters of the six *P. sinensis* populations are summarized in Table 6. Na ranged from 4.250 to 8.500, Ho ranged from 0.375 to 0.572, He ranged from 0.444 to 0.648, and the I ranged from 0.873 to 1.475. The results indicate that the CY population exhibited the highest genetic diversity, while the HH population had the lowest. Furthermore, the Fis values for all populations were greater than zero, suggesting a certain degree of heterozygote deficiency (Table 6).

### 3.4. Genetic Differentiation Analysis Among P. sinensis Population

An analysis of genetic variation among the six *P. sinensis* populations was conducted using AMOVA (Table 7). The results showed that genetic differentiation primarily occurred within individuals, accounting for 67% of the total variation, followed by variation among individuals within populations (19%) and variation among populations (14%). All sources of variation were statistically significant (*p* < 0.05). The estimated gene flow (*Nm*) was 1.481, which is greater than 1, indicating substantial gene exchange among populations. As shown in Table 8, most population pairs exhibited moderate genetic differentiation and moderate gene flow (0.05 ≤ F_ST_ < 0.15, 1 ≤ Nm < 4). The highest genetic differentiation coefficient was observed between the HH and HS populations (F_ST_ = 0.190), while the lowest was found between the CY and DT populations (F_ST_ = 0.022). Notably, the genetic differentiation coefficients among the CY, W, and DT populations were all below 0.05, with exceptionally strong gene flow among them (Nm > 4). Nei’s genetic distance, which measures genetic divergence between populations, is presented in Table 9. The HS population has the greatest genetic distance from the HH and JP populations, while the DT population is most closely related to the CY population.

### 3.5. Population Genetic Structure Analysis and Germplasm Identification of Markers

**Figure 4:**
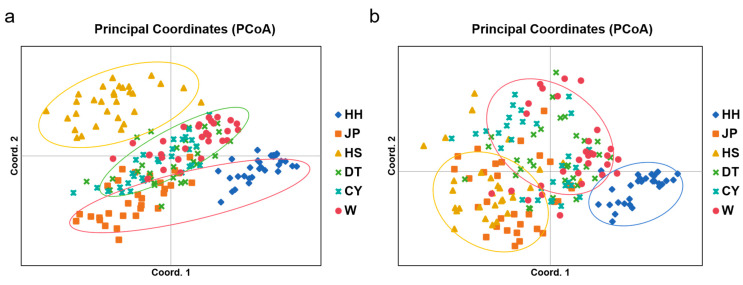
Principal coordinate analysis (PCoA) based on 12 SSR loci for 180 individuals from six *P. sinensis* populations [Table S1]. The colors and shapes represent the distribution of the populations: HH in blue, JP in orange, HS in yellow, DT in green, CY in light blue, and W in red. The axes of the plot are labeled with the principal components and the percentage of variance explained, as shown in (**a**,**b**).

**Figure 5:**
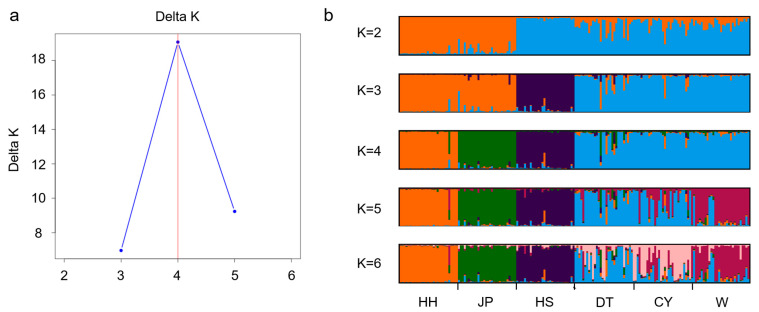
Population structure analysis based on 12 SSR loci. (**a**). Delta K plot from the STRUCTURE analysis. The Delta K value reaches its maximum when K = 4; (**b**). Population structure analysis of the six Chinese soft-shelled turtle populations using STRUCTURE, with K values ranging from 2 to 6.

The three principal components from the PCoA analysis explained 26.12% of the total genetic variation (Principal Component 1, 11.36%; Principal Component 2, 7.94%; and Principal Component 3, 6.82%). A plot based on Principal Components 1 and 2 separated the populations into three distinct groups: the HS population formed its own cluster; the HH and JP populations clustered together; and the W, CY, and DT populations were grouped together. A plot based on Principal Components 1 and 3 revealed distinct population clustering patterns, with the HH population forming a separate group; the HS and JP populations clustering together; and the W, CY, and DT populations grouped into another cluster (Figure 4). The results of the STRUCTURE genetic analysis indicated that when *K* = 4, the Delta *K* values reached their maximum (Figure 5a), suggesting that the six populations could be divided into four subgroups. Specifically, the HH, JP, and HS populations each formed distinct clusters, while the DT, CY, and W populations were grouped together (Figure 5b).

**Figure 6:**
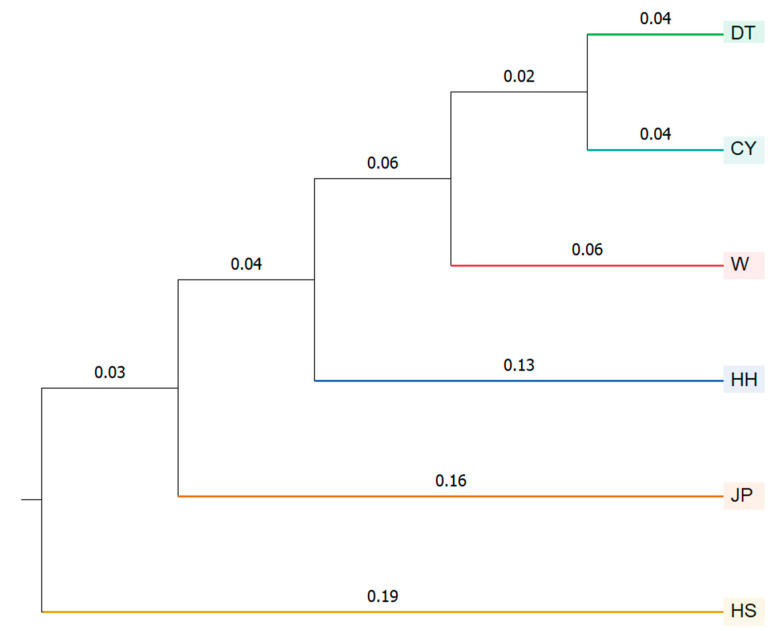
UPGMA dendrogram of six *P. sinensis* populations based on Nei’s genetic distance using 12 polymorphic SSR loci. The DT and CY populations first clustered together, followed by W, then JP, and subsequently HH, with HS forming the final distinct cluster.

**Figure 7:**
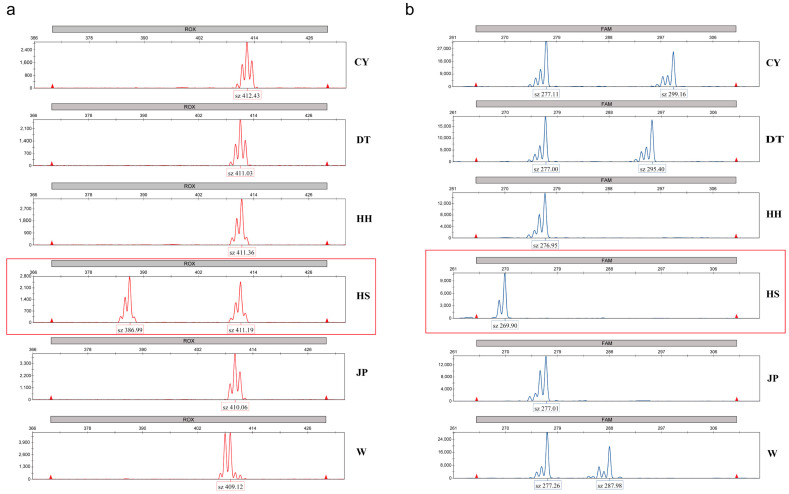
Capillary electrophoresis results of six *P. sinensis* populations. The *X*-axis represents fragment size in base pairs (bp), while the *Y*-axis displays the fluorescence intensity of the amplification products. Each population corresponds to a distinct electropherogram, and based on fragment size differences, the HS population can be differentiated from the other populations, the location marked in red is the typing result of the HS population at that locus. (**a**) SSR marker no. 3 and (**b**) SSR marker no. 8.

A population clustering analysis using Nei’s genetic distance was performed, and a more detailed population structure was observed in the UPGMA tree constructed using MEGA: DT and CY first clustered together, then grouped with W, followed by HH clustering with the other populations, and finally grouping with JP. The HS population was the first to separate from the others (Figure 6). Based on differences in banding patterns, two molecular markers, specifically loci 3 and 8 in Supplementary Table S1, were identified that can distinguish the HS population from the other populations (Figure 7). According to the results from Figure 5b, the genotypes of the HS population at loci 3 and 8 show distinct differences compared to the other populations. At locus 3, the HS population exhibited clear fluorescence peaks at 386.99 bp and 411.19 bp, while the other populations showed peaks near 411 bp. At locus 8, the HS population displayed a fluorescence peak around 269 bp, whereas the other populations exhibited peaks at positions greater than 276 bp (Supplementary Table S4).

## 4. Discussion

Morphological analysis is one of the effective methods for studying genetic variation within species [27]. In this study, cluster analysis based on morphological traits revealed that the JP and HH populations were grouped together; the DT and CY populations first clustered together and then with the W population; and, finally, the remaining populations clustered with the HS population. This result is consistent with the UPGMA tree analysis, where there is a close correlation between morphological development and genetic relationships. Discriminant analysis based on morphological traits was conducted for the six populations, and a discriminant function was established, resulting in an overall discriminant accuracy of 71.4%. The highest accuracy was observed in the DT and HH populations. Some individuals from the JP population were misclassified as belonging to the HH or HS populations, while a significant proportion of the CY and W populations were misclassified as DT and HH populations. The results of the cluster and discriminant analyses indicate a certain morphological similarity between the HH, JP, and HS populations, while the DT, CY, and W populations showed minor morphological differences. This morphological similarity also suggests a potential phylogenetic relationship between the populations [28]. Additionally, two microsatellite markers were identified in this study that can be used to distinguish the HS population, providing a reference for future germplasm identification in conjunction with the discriminant function.

Higher genetic diversity within a species increases its adaptability to environmental changes and enhances its evolutionary potential, making the study of genetic diversity a foundational aspect of breeding research [29]. In this study, 12 highly polymorphic SSR markers with PIC values ranging from 0.033 to 0.891, He values between 0.032 and 0.801, and Na values ranging from 1.833 to 12.000 were developed based on Super-GBS sequencing to analyze the genetic diversity of six *P. sinensis* populations. Compared to the 15 SSR markers (Na range: 2–7) developed by Que [30], the SSR markers developed in this study exhibit higher polymorphism.

Genetic diversity parameters such as Na, Ne, Ho, and He reflect the genetic variation within and between populations or individuals. Among these, He is a key indicator of genetic diversity, as it is calculated based on allele frequencies and is less affected by sample size, providing a reliable measure of a population’s genetic diversity [31]. In this study, the CY population exhibited the highest genetic diversity, while the HH population had the lowest. The genetic diversity of the JP population was higher than that of the HH population, which is consistent with the findings of Gang [8]. In contrast, Zhang [32] studied several *P. sinensis* varieties using RAPD technology and concluded that the JP population had higher genetic diversity than the W population. However, this study reached the opposite conclusion, demonstrating that SSR markers provide greater stability and specificity compared to RAPD. The observed heterozygosity (Ho) ranged from 0.375 to 0.572, while the expected heterozygosity (He) was consistently higher than Ho. Additionally, the Fis values for all populations were greater than zero, indicating the presence of heterozygote deficiency. This phenomenon may be attributed to factors such as artificial selection, inbreeding, or small population sizes.

Genetic differentiation among six *P. sinensis* populations was analyzed based on 12 microsatellite loci. The results of the AMOVA analysis revealed significant genetic variation among populations (*p* < 0.05) and moderate gene flow (1 < Nm < 4). The majority of genetic variation was observed within individuals, whereas Zhang [32] reported that genetic variation in *P. sinensis* primarily occurs within populations. Moderate gene flow was generally detected among populations (1 ≤ Nm < 4), with particularly strong gene exchange among the DT, W, and CY populations (4 < Nm). Furthermore, the Hardy–Weinberg equilibrium analysis revealed significant deviations from equilibrium at multiple SSR loci across all populations. Comparatively, the HH population exhibited lower genetic diversity and relatively weaker gene exchange with the other populations. The primary habitat of the HH population is located in the Yellow River Basin [33], and its geographic isolation from the other populations likely restricts gene flow. The CY population, originally from Changyong Village in Hengyang County, Hunan Province, was formed by local villagers who spontaneously bred wild *P. sinensis*. The W population was artificially selected from color-varied individuals of the DT population, forming a new variety [17]. Therefore, both the CY and W populations have undergone a certain degree of artificial selection. The three populations exhibited highly frequent gene flow (*Nm* > 4). While frequent gene exchange enhances genetic diversity, imbalanced migration rates and limited effective population sizes may lead to genetic drift, ultimately causing deviations from equilibrium. Fis is a key indicator for assessing genetic dynamics within a population. A positive Fis value (Fis > 0) suggests a deficiency of heterozygotes, whereas a negative Fis value (Fis < 0) indicates an excess of heterozygotes [34]. In this study, the Fis values for all populations were greater than zero. Among the 12 SSR markers analyzed, 4 exhibited negative Fis values, while the remaining markers had positive values, indicating the presence of heterozygote deficiency in *P. sinensis* populations. Despite the occurrence of frequent gene flow among populations, heterozygote deficiency persisted, which may be attributed to inbreeding, artificial selection pressure during aquaculture, or genetic drift resulting from small population sizes.

The F_ST_ is an important indicator for measuring the degree of genetic differentiation among populations or subpopulations, and it is negatively correlated with gene flow. According to Wright [35], when F_ST_ ≥ 0.05, it indicates moderate genetic differentiation between populations. From the results in Table 5, except for the F_ST_ between the CY, W, and DT populations, which is less than 0.05, all other population pairs showed F_ST_ values greater than 0.05. This suggests that, despite frequent gene flow, most populations still exhibit moderate genetic differentiation. AMOVA analysis also revealed significant genetic variation among populations. However, the excessively frequent gene exchange between the CY, W, and DT populations has suppressed genetic differentiation between these groups. The magnitude of genetic distance is an important factor in studying population genetic diversity and is positively correlated with population divergence time. Analysis of the genetic distances between populations showed that the HS population had the greatest genetic distance from the HH and JP populations, while the DT and CY populations exhibited the smallest genetic distance. Crossbreeding between populations with greater genetic distances is more likely to result in heterosis, which can provide valuable insights for future hybrid breeding studies [36].

The STRUCTURE analysis divided the six *P. sinensis* populations into four subgroups. In the phylogenetic tree analysis, the DT population clustered with the CY population first, followed by the W population, then the JP and HH populations, and finally the HS population. This clustering pattern is consistent with the morphological clustering analysis results. In the PCoA results, the HS population was consistently assigned to a separate cluster. Based on the integrated findings, the HS population was classified as one group; HH and JP as another; and DT, W, and CY as a third group. This classification aligns with the results of previous studies conducted in our laboratory and is consistent with similar conclusions drawn by Gang [8]. The HH, DT, and HS populations represent the native *P. sinensis* of China. The JP population, on the other hand, is a new variety that was developed after multiple generations of selective breeding from the native *P. sinensis* imported from Japan [17]. The fact that the HH and JP populations clustered together may be attributed to the Japanese population’s origin from the Chinese *P. sinensis*, which was historically introduced as food to Japan, followed by long-term geographic isolation. The Yellow River *P. sinensis*, native to the Yellow River Basin, is geographically close to Japan, and it is hypothesized that the Japanese population originated from the Yellow River *P. sinensis* and underwent genetic differentiation due to long-term isolation. However, despite this geographic proximity, the two populations have undergone substantial genetic differentiation (0.15 < F_ST_) [33]. There has been no prior research specifically on the HS population, which originates from Guangxi. The *P. parviformis* is a type of turtle from Quanzhou in Guangxi. Some studies classify the “small turtle” as a subspecies of *P. sinensis*, while others consider it a subspecies without reproductive isolation from *P. sinensis* [37]. In this study, the HS population was distinguished from the other populations and categorized into a separate subgroup. Given that both the HS population and the *P. parviformis* are distributed in the Guangxi region and are geographically close, it is plausible to hypothesize that there might be a phylogenetic relationship between the HS population and the *P. parviformis*. This hypothesis, however, requires further investigation. Additionally, all samples collected in this study were from artificially cultured populations, thus introducing certain limitations to the research. Future studies should include a greater number of wild samples to enhance the comprehensiveness and robustness of the findings.

## 5. Conclusions

This study established a discriminant function based on morphological data, achieving a classification accuracy of 71.4%. Additionally, two SSR markers capable of distinguishing the HS population were developed. By integrating morphological and genetic analyses, the six *P. sinensis* populations were classified into three subgroups. Genetic variation was primarily observed within individuals, with significant genetic differentiation detected among most populations. Frequent gene exchange was observed among the CY, W, and DT populations, which increased genetic diversity within these groups. However, this frequent gene flow also suppressed genetic differentiation between them. In future studies, it is recommended to impose appropriate restrictions on population exchanges within the CY, W, and DT cultured groups. While expanding the breeding populations, care should be taken to avoid inbreeding, thereby ensuring the conservation of *P. sinensis* germplasm resources. The results of this study provide a clear understanding of the genetic structure, differentiation, and diversity of the six major breeding populations of *P. sinensis*. Moreover, the established discriminant function and the developed SSR markers for germplasm identification can offer a theoretical basis for the conservation of genetic resources in the *P. sinensis* aquaculture industry. 

## Figures and Tables

**Table 1:** Sampling information of six different populations of *P. sinensis*.

Population	Collection Date	Number	Longitude and Latitude
Dongting (DT)	25 November 2021	50	29°5′7.94″ N, 111°47′12.55″ E
Changyong (CY)	7 November 2021	30	26°54′53.60″ N, 112°24′59.62″ E
Black (W)	13 November 2021–11 June 2023	40	29°7′43.90″ N, 119°25′58.91″ E
Yellow River (HH)	25 November 2021–22 April 2023	30	36°50′52.30″ N, 116°53′7.33″ E
Japanese (JP)	13 November 2021	30	27°3′4.86″ N, 116°37′15.02″ E
Huangsha (HS)	13 November 2021	30	23°36′43.85″ N, 110°6′49.68″ E

**Table 2:** Explanation of codes and corresponding full names.

ID	Figure Code	Abbreviation	Full Name
X1	1–2/1–3	BL/CL	Body Length/Carapace Length
X2	8–9/1–3	SH/CL	Shell Height/Carapace Length
X3	6–7/1–3	CW/CL	Carapace Width/Carapace Length
X4	10–11/1–3	PL/CL	Plastron Length/Carapace Length
X5	4–5/1–3	PW/CL	Plastron Width/Carapace Length
X6	12–3/1–3	BAW/CL	Back Apron Width/Carapace Length

**Table 3:** Discriminant analysis results of the six *P. sinensis* populations.

Project	Population	HH	HS	JP	DT	CY	W	Total	Comprehensive Discriminant Rate %
Initial	HH	22	0	5	1	0	2	30	71.8
	HS	1	20	5	3	0	1	30	
	JP	4	3	20	2	1	0	30	
	DT	1	0	0	45	1	3	50	
	CY	4	2	1	5	18	0	30	
	W	5	0	2	5	2	26	40	
Discriminant accuracy %		73.1	66.7	66.7	90.0	60.0	65.0		
Cross-validation	HH	22	0	5	1	0	2	30	71.4
	HS	1	20	5	3	0	1	30	
	JP	4	2	20	2	1	1	30	
	DT	1	0	0	45	1	3	50	
	CY	3	2	1	7	17	0	30	
	W	5	0	2	5	2	26	40	
Discriminant accuracy %		73.1	66.7	66.7	90.0	56.7	65.0		

**Table 4:** Genetic diversity parameters of the 12 SSR loci.

Locus	Na	Ne	Ho	He	I	PIC	Nm	Fna	Fis
1	11.167	5.406	0.750	0.746	1.831	0.891	1.213	0.020	−0.005
2	11.333	4.777	0.994	0.780	1.849	0.831	2.949	0.000	−0.275
3	7.833	4.179	0.450	0.692	1.529	0.836	1.102	0.150	0.349
4	10.000	5.014	0.553	0.791	1.834	0.825	3.825	0.138	0.302
5	3.167	2.014	0.177	0.483	0.805	0.461	1.651	0.215	0.635
6	1.833	1.036	0.022	0.032	0.081	0.033	9.295	0.035	0.308
7	3.333	1.791	0.051	0.405	0.688	0.386	2.146	0.468	0.875
8	12.000	4.758	0.711	0.670	1.707	0.855	0.863	0.004	−0.061
9	3.333	1.737	0.297	0.368	0.662	0.394	1.499	0.092	0.195
10	3.667	2.086	0.259	0.450	0.833	0.525	0.780	0.492	0.424
11	6.500	3.040	0.502	0.650	1.317	0.755	1.227	0.096	0.228
12	9.500	5.409	0.835	0.801	1.847	0.813	6.114	0.016	−0.042
Average	6.972	3.437	0.467	0.572	1.249	0.634	1.213	0.144	−0.005

Note: No. of different alleles (Na); No. of effective alleles (Ne); expected heterozygosity (He); observed heterozygosity (Ho); Shannon Index (I); polymorphism information content (PIC); number of migrants per generation (Nm); frequency of null alleles (Fna); inbreeding coefficient (Fis).

**Table 5:** A test for deviations from Hardy–Weinberg equilibrium.

Group		Locus											
		Y1	Y2	Y3	Y4	Y5	Y6	Y7	Y8	Y9	Y10	Y11	Y12
HH	Prob	0.118	0.033	0.000	0.001	0.018	-	0.000	0.171	0.448	-	0.481	0.001
	Signif	ns	*	***	***	*	-	***	ns	ns	-	ns	***
JP	Prob	0.173	0.675	0.101	0.000	0.000	0.926	0.000	0.010	0.045	0.000	0.000	0.430
	Signif	ns	Ns	ns	***	***	ns	***	**	*	***	***	ns
HS	Prob	0.068	0.578	0.392	0.002	0.000	0.926	0.000	0.000		0.000	0.020	0.965
	Signif	ns	Ns	ns	**	***	ns	***	***		***	*	ns
DT	Prob	0.774	0.102	0.089	0.000	0.000	-	0.000	0.967	0.000	0.000	0.331	0.000
	Signif	ns	Ns	ns	***	***	-	***	ns	***	***	ns	***
CY	Prob	0.734	0.507	0.789	0.157	0.006	-	0.000	0.356	0.000	0.000	0.150	0.000
	Signif	ns	Ns	ns	ns	**	-	***	ns	***	***	ns	***
W	Prob	0.000	0.222	0.000	0.673	0.000	0.000	0.000	0.669	0.004	0.000	0.021	0.003
	Signif	***	Ns	***	ns	***	***	***	ns	**	***	*	**

“ns” indicates that the locus is in Hardy–Weinberg equilibrium (*p* ≥ 0.05); “*” denotes a significant deviation from Hardy–Weinberg equilibrium (*p* < 0.05); “**” indicates a highly significant deviation from Hardy–Weinberg equilibrium (*p* < 0.01); “***” represents an extremely significant deviation from Hardy–Weinberg equilibrium (*p* < 0.001); “-” refers to only one genotype at this locus in the population, without this value.

**Table 6:** Genetic diversity analysis of six *P. sinensis* populations based on 12 SSR Loci.

Pop	Na	Ne	Ho	He	I	Fis
DT	8.500	4.019	0.499	0.648	1.453	0.230
CY	8.333	4.221	0.572	0.646	1.475	0.115
W	8.083	4.225	0.488	0.636	1.451	0.233
HH	4.250	2.472	0.431	0.444	0.873	0.029
JP	6.167	2.900	0.436	0.558	1.162	0.219
HS	6.500	2.787	0.375	0.503	1.076	0.254

**Table 7:** Analysis of molecular variance (AMOVA) from six *P. sinensis* populations.

Source	*df*	*SS*	MS	Est. Var.	%	Nm
Among populations	5	201.178	40.236	0.599	14 *	-
Among individuals within populations	174	750.967	4.316	0.770	19 *	
Within individuals	180	499.500	2.775	2.775	67 *	-
Total	359	1451.644		4.144	100	1.481

Note: *df*, degrees of freedom; *SS*, sum of squares; MS, mean square; Est. Var., estimated variance; Nm, gene flow; “*” indicates that the difference is statistically significant (*p* < 0.05).

**Table 8:** Genetic differentiation and gene flow among six *P. sinensis* populations.

Population	HH	JP	HS	DT	CY	W
HH	-	0.158	0.190	0.094	0.116	0.089
JP	1.333	-	0.114	0.064	0.063	0.094
HS	1.067	1.938	-	0.080	0.075	0.089
DT	2.415	3.627	2.873	-	0.022	0.029
CY	1.902	3.700	3.076	11.325	-	0.037
W	2.550	2.416	2.562	8.342	6.465	-

Note: Gene flow (Nm) is below the diagonal line; genetic differentiation (F_ST_) is above the diagonal line.

**Table 9:** Nei’s genetic distance among six *P. sinensis* populations.

Population	HH	JP	HS	DT	CY	W
HH	-					
JP	0.436	-				
HS	0.593	0.430	-			
DT	0.225	0.243	0.288	-		
CY	0.293	0.254	0.280	0.081	-	
W	0.247	0.368	0.325	0.115	0.139	-

## Data Availability

The data that support the findings of this study are openly available from the NCBI at https://www.ncbi.nlm.nih.gov/sra/PRJNA1053618 (accessed on 29 May 2024), reference number PRJNA1053618.

## Supplementary Materials

The following supporting information can be downloaded at https://www.mdpi.com/article/10.3390/genes16030318/s1, Table S1: Information of the SSR primers analyzed; Table S2: Morphological metrics of six different *Pelodiscus sinensis* populations; Table S3: Proportion parameters of different characters in different populations of *P. sinensis*; Table S4: The genotyping results of 12 SSR markers for 180 *P. sinensis* samples (individuals × loci).
