# Supplementary material for: Research on SSR Genetic Molecular Markers and Morphological Differences of Different Pelodiscus sinensis Populations
Source: Genes (Basel). 2025 Mar 7;16(3):318. doi: 10.3390/genes16030318 (PMC11942387; doi:10.3390/genes16030318)
Supplement: Supplementary file 1 [file genes-16-00318-s001.zip › Table S1.pdf]

Table S1. Information of the SSR primers analyzed.

Locus	Repeat motif	Annealing temperature/℃	Fluorescent dye	Fragment/bp	Primer sequence
1	(T)19	61	FAM	300-334	F：ACTATGCAGAGGTAGGCACCACTC<br>R：AGAGGCCTTGAAGGAGAGTTTCAT
2	(T)15	58	FAM	430-453	F：GAAGAAAGTACCCGGAACTGCTTT<br>R：CTTGTTTCCATCCTCCAAAATCAC
3	(T)15	57	ROX	385-415	F：CTGAAG TCTGGCACTAAGCTTTCC<br>R：GTCTAAATTCCGAAGCGGGTAAAA
4	(T)15	62	HEX	402-421	F：GGTCAGTTCCCTATGCACTGTCTT<br>R：TAAGTTGGGGACTGCCTGTATGTT
5	(GA)6	59	ROX	264-267	F：GGCACAGAGAGAACAATATTGCCT<br>R：TCTCCTGATCCTTGTTGTCCTTTC
6	(TCC)6	58	FAM	421-426	F：CTGCACTGGTGAGTTCTTTAGCAA<br>R：GGATCAGAACACAGATCAGCAGAA
7	(AG)6	59	HEX	458-482	F：TAACTCAGTTCATGCCAAAGTTGC<br>R：GGTTTTAACCTTGCAGCTCTGAAA
8	(AC)10	61	FAM	269-301	F：TAGCCCCGAGATTATCAGAGACAG<br>R：TGTGGCTGGTTGGTGTAGTAAGAA
9	(TG)9	57	ROX	233-240	F：GTGATGGAGTACAGGGGGAGTG<br>R：GTACCCCTTGCTGTTCGTTAAATG
10	(TG)6	61	HEX	381-408	F：TGCTGAGGAGAGTAGAGGAGAAA<br>R：CCATCGAAGAGAAAATGAGCATCT
11	(GCA)7	60	HEX	412-432	F：GTTCATCAGTATCATCGCCTCCTT<br>R：AGCCAGTGAGAACTGTTGAGTGTG
12	(CTTTC)7	60	TAMRA	392-428	F：TCTGAAACATTGCCTCTTTCTTCC<br>R：GCACAGAGACACTGCATGAGTTTT
