# Supplementary material for: Research on SSR Genetic Molecular Markers and Morphological Differences of Different Pelodiscus sinensis Populations
Source: Genes (Basel). 2025 Mar 7;16(3):318. doi: 10.3390/genes16030318 (PMC11942387; doi:10.3390/genes16030318)
Supplement: Supplementary file 1 [file genes-16-00318-s001.zip › Table S2.pdf]

Table S2. Morphological metrics of six different *Pelodiscus sinensis* populations.

Group	Weight(g)	Body Length(mm)	Carapace Length(mm)	Plastron Width(mm)	Shell Height(mm)	Plastron Length(mm)	Carapace Width(mm)	Back Apron Width(mm)
Dongting (DT)	755.07±28.05	139.40±8.27	175.13±5.48	115.60±4.39	57.82±3.31	132.42±4.32	138.23±5.40	23.83±2.63
Changyong (CY)	620.33±44.33	131.78±7.10	154.82±9.52	123.95±15.52	51.52±4.07	120.66±7.12	122.17±11.40	21.92±2.07
Black (W)	891.30±293.56	149.21±17.95	177.23±14.71	119.16±11.45	61.34±7.45	139.98±12.36	141.19±15.33	23.25±3.75
Yellow river (HH)	677.84±103.62	124.93±13.71	164.18±16.23	111.97±10.77	51.05±3.67	131.14±7.51	127.50±8.99	24.82±4.49
Japanese (JP)	857.40±198.23	141.60±10.67	178.51±10.55	121.97±7.34	59.38±5.65	134.84±7.91	150.06±8.18	28.15±2.70
Huangsha (HS)	826.98±181.13	136.60±12.62	178.78±12.96	119.61±8.93	62.26±5.34	135.04±9.79	151.90±13.86	30.30±3.38
