# Supplementary material for: Research on SSR Genetic Molecular Markers and Morphological Differences of Different Pelodiscus sinensis Populations
Source: Genes (Basel). 2025 Mar 7;16(3):318. doi: 10.3390/genes16030318 (PMC11942387; doi:10.3390/genes16030318)
Supplement: Supplementary file 1 [file genes-16-00318-s001.zip › Table S3.pdf]

Table S3. Proportion parameters of different characters in different populations of *P. sinensis*.

Group	X1	X2	X3	X4	X5	X6
HH	0.757±0.050	0.680±0.046	0.307±0.021	0.792±0.034	0.772±0.024	0.150±0.016
HS	0.763±0.039	0.671±0.044	0.349±0.026	0.756±0.020	0.840±0.027	0.170±0.019
JP	0.750±0.033	0.686±0.032	0.313±0.025	0.767±0.030	0.817±0.029	0.141±0.018
DT	0.800±0.025	0.650±0.020	0.336±0.025	0.747±0.021	0.783±0.027	0.143±0.016
CY	0.808±0.018	0.719±0.088	0.304±0.029	0.745±0.047	0.766±0.073	0.146±0.017
W	0.839±0.049	0.676±0.033	0.342±0.042	0.793±0.028	0.803±0.045	0.128±0.016
